# Health Tract, No. 47

**Published:** 1865

**Authors:** 


### HEALTH TRACT, No. 47.

---

### THE THREE P'S.

**PROMPTITUDE, PERSEVERANCE, AND PAINSTAKING.**

At the close of the last century, a poor, awkward, uncouth boy entered London, but he was so long, lank, and ungainly, that he seemed fit only to be the drudge of a printing-office; run errands, bring water, sweep the floor, and the like. Already had poverty and the hardness of the world made him sour, unhopeful, and despondent. Under less discouragements, many a youth has abandoned himself to an aimless life, having no higher aim than to live but for the day; or, worse still, has plunged headlong into all the extravagances and indulgences connected with thriftlessness and crime. But the boy had vigorous health; this imparted to him a mental vim, a moral power, which soon showed itself to his employer. He was prompt, persevering, and painstaking; and with these three qualities, in spite of the fact that he was good at nothing, in every thing tolerable only, he made his patient way, step by step, to the woolsack of England, and lately died, (worth a million of dollars,) among the most honored men of his nation and age—Lord Chief-Justice Campbell. In this case, vigorous health was a mine of wealth; a better fortune than if he had been the heir of many thousands. And certain is it, that the world would be a happier world, and the men in it would be happier, better, and greater, if one tithe of the time, and care, and study which parents bestow on the accumulation of money to leave to their children, were devoted to the physical education and training necessary to secure a vigorous constitution. Of any two young men starting on the race of life, one poor but healthy, the other rich and effeminate, other things being equal, the chances for usefulness, honor, and a well-remembered name, are manifold in favor of the former. Who that reads this article will lay it down and resolve: "I will do more to leave to my children a vigorous constitution?"

Another element in the success of Lord Chief Justice Campbell was, that his employer seeing his dull nature, but noticing at the same time that when he had any thing to do, he went at it promptly, and with great painstaking kept at it until the work in hand was done, although done painfully slow, he patted him on the shoulder, always spoke cheerfully to him, and thus stimulated him to greater activities. How many a youth at school, how many an apprentice in the shop, how many a child in the family, has gone out in the night of a blighted life, who, with humane encouragements, might have lived usefully and died famous, let the passionate teacher and master and parent inquire, and do a little more patting on the shoulder.

